# Correlational analysis of three-dimensional spinopelvic parameters with standing balance and gait characteristics in adolescent idiopathic scoliosis: A preliminary research on Lenke V

**DOI:** 10.3389/fbioe.2022.1022376

**Published:** 2022-11-30

**Authors:** Yanan Liu, Xianglan Li, Xiaoran Dou, Zhiguan Huang, Jun Wang, Bagen Liao, Xiaohui Zhang

**Affiliations:** ^1^ Department of Sports Medicine, Guangzhou Sport University, Guangzhou, China; ^2^ School of Sports and Health, Guangzhou Sport University, Guangzhou, China; ^3^ Gosun Medical Imaging Diagnosis Center of Guangdong Province, Guangzhou, China

**Keywords:** adolescent idiopathic scoliosis, standing balance, 3d parameters, gait, correlation analysis

## Abstract

**Background:** Adolescent idiopathic scoliosis (AIS), the most common spinal deformity, possibly develops due to imbalanced spinal loading following asymmetric development. Since altered loading patterns may affect standing balance and gait, we investigated whether a correlation exists between balance ability, gait pattern, and the three-dimensional radiographic spinopelvic parameters in AIS patients.

**Methods:** A cross-sectional observational study was conducted with 34 AIS patients (aged 10–18 years) and an equal number of healthy age and sex-matched teenagers (normal group). We obtained the spinopelvic three-dimensional parameters and balance parameters simultaneously through the EOS imaging system and gait and center of pressure (CoP) characteristics using a plantar pressure measurement mat. Besides determining the intergroup differences in balance and gait parameters, multiple linear regression analyses were performed to identify any correlation between the static plantar pressure and radiographic parameters.

**Results:** Compared to the normal group, the CoP_x_ is lower, the CoP path length and 90% confidence ellipse area were significantly higher in AIS patients (AIS: −13.7 ± 5.7 mm, 147.4 ± 58.1 mm, 150.5 ± 62.8 mm^2^; normal: −7.0 ± 5.4 mm, 78.8 ± 32.0 mm, 92.1 ± 41.7 mm^2^, respectively), correlated with apical vertebra translation, sagittal pelvic tilt, and pelvis axial rotation, respectively. Moreover, AIS patients had a shorter stance phase (61.35 ± 0.97 s vs. 62.39 ± 1.09 s), a longer swing phase (38.66 ± 0.97 s vs. 37.62 ± 1.08 s), and smaller maximum pressure peaks in the gait cycle, especially on the left foot, as compared to healthy subjects. Moreover, the CoP trajectory in AIS patients was different from the latter, and changes in the bipedal trend were not consistent.

**Conclusion:** The standing balance and gait characteristics of AIS patients are different from those of healthy subjects, as reflected in their three-dimensional spinopelvic radiographic parameters. Trial registration: The study protocol was registered with the Chinese Clinical Trial Registry (Number ChCTR1800018310) and the Human Subject Committee of Guangzhou Sport University (Number: 2018LCLL003).

## Introduction

Scoliosis, a three-dimensional deformity of the spine, is characterized by a lateral curvature of ≥10° in the spine in the coronal plane (Altaf et al., 2013), with adolescent idiopathic scoliosis (AIS) being the most common variety encountered in routine pediatric and orthopedic practice (Lonstein et al., 1994). Epidemiological studies show that although 1%–3% of all children between 10 and 16 years of age experience varying degrees of spinal curvatures, most of them do not require cautious intervention (Weinstein et al., 2008). While the life expectancy of AIS patients is not significantly different from the general population (Pehrsson et al., 1992), their quality of life is affected by several multisystem consequences of the deformity, including reduced respiratory function (Koumbourlis et al., 2006), back pain (Wong et al., 2019), degenerative spine disorders (Weinstein et al., 2003), and concerns relating to their body image (Gallant et al., 2018) and mental health (Mitsiaki et al., 2022). Therefore, holistic management strategies for AIS are gradually becoming the focus of research.

Structurally, scoliosis is not only caused by the lateral deviation of the vertebrae but involves a three-dimensional malalignment of the vertebrae leading to geometric and morphological changes in the trunk and pelvis (Cheng et al., 2015). Although the pathogenesis of the malalignment in AIS is still unknown (Lowe et al., 2000) (Kikanloo et al., 2019), existing studies suggest the role of abnormal balance (Nault et al., 2002). Due to the high susceptibility of adolescents to scoliosis, great attention is given to balance and gait disorders in this age group (Mahaudens et al., 2009) (Daryabor et al., 2016) (Yang et al., 2013) (Karimi et al., 2016) (Quervain et al., 2004). It is known that optimal balance is essential for performing both simple everyday tasks and complex movement patterns, such as walking (Saggini et al., 2021). Accordingly, balance dysfunctions can lead to postural instability, walking difficulty, and even, falls. Recent evidence suggests a potential correlation between different gait and balance parameters in AIS patients and that the spine and pelvis play a key role in regulating body balance.

The traditional research on AIS has focused on coronal plane malalignments; fortunately, recent advances in the field of radiography have shifted the attention toward sagittal and axial plane involvements, leading to notable discoveries (Nolte et al., 2020). A balanced three-dimensional spinal alignment serves as a crucial biomechanical foundation to enable flexibility during spinal movements and postural stability while carrying axial loads. Previous studies have reported diminished balance abilities in patients with AIS(Wiernicka et al., 2019) (Beaulieu et al., 2008). Although a few authors have described a relationship between the spinal deformity status and static balance, all these reports studied the deformity from a single plane rather than using a three-dimensional perspective. To the best of our knowledge, no study has explored the association between the balance and imaging acquisition parameters in these patients.

Therefore, the present study aimed to analyze the differences in the balance ability between AIS patients and their age and sex-matched normal individuals (normal group), and whether a correlation exists between their three-dimensional spinopelvic parameters and static balance acquired simultaneously. A secondary objective of the study was to compare the gait characteristics of AIS patients with the normal group subject. We hypothesized that the static balance of AIS patients will be different from that of the normal group subjects.

## Materials and methods

### Ethics clearance

The study was conducted in accordance with the principles of the Declaration of Helsinki. The study protocol was approved by the Human Subject Committee of Guangzhou Sport University (Number: 2018LCLL003) and the trial was registered with the Chinese Clinical Trial Registry (Number: ChCTR1800018310). The parents or legal guardians of all participants provided informed consent for participating in the study.

### Sample size

Using the G*Power software (Germany G*Power version 3.1), we calculated the sample size as 34 using an effect size with a medium value of 0.35, an alpha probability of 0.05, and a beta probability of 0.95.

### Participants

We enrolled patients aged 10–18 years diagnosed with AIS by an orthopedist between January 2021 and April 2022 in this study. The patients were included if the diagnosis of AIS was consistent with the curve classification of Lenke V (Slattery and Verma et al., 2018), Cobb’s angle between 10° and 40°, and were skeletally immature (defined as 0–3 on the Risser’s sign (Wittschieber et al., 2012)). Those with non-idiopathic types of scoliosis (such as neuromuscular or congenital), having a history of treatment with brace or other modalities, or any other musculoskeletal alterations or orthopedic history, a lower limb discrepancy of >1 cm, or malignant and psychiatric diseases, were excluded from the study. An equal number of age and sex-matched healthy teenagers were recruited from the same city as the “normal group” through advertising.

### Procedure

**FIGURE 1:**
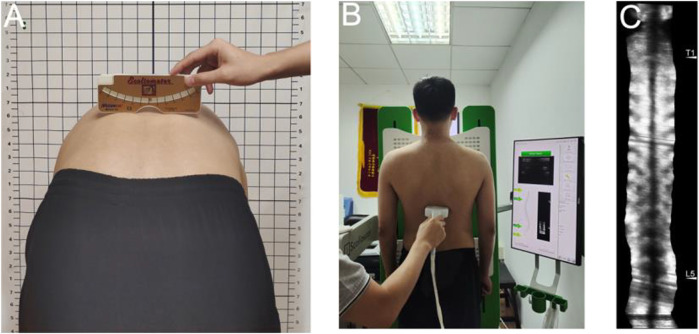
Scoliosis screening methods-**(A)** Adam’s test and trunk rotation angle measured with a Scoliometer; **(B)** A radiation-free spine ultrasound imaging system; **(C)** Ultrasound imaging.

Demographic information for every participant was collected. For healthy subjects, we used scoliosis screening methods, including Adam’s test, trunk rotation angle measured with a scoliometer, and a radiation-free spine ultrasound imaging system (Scolioscan^®^ SCN801, Hong Kong) to ensure there were no scoliotic deformities (Figure 1). Spinal ultrasonography has been shown to have excellent reliability in the diagnosis of mild to moderate scoliosis (Lv et al., 2019) (Ungi et al., 2020).

**FIGURE 2:**
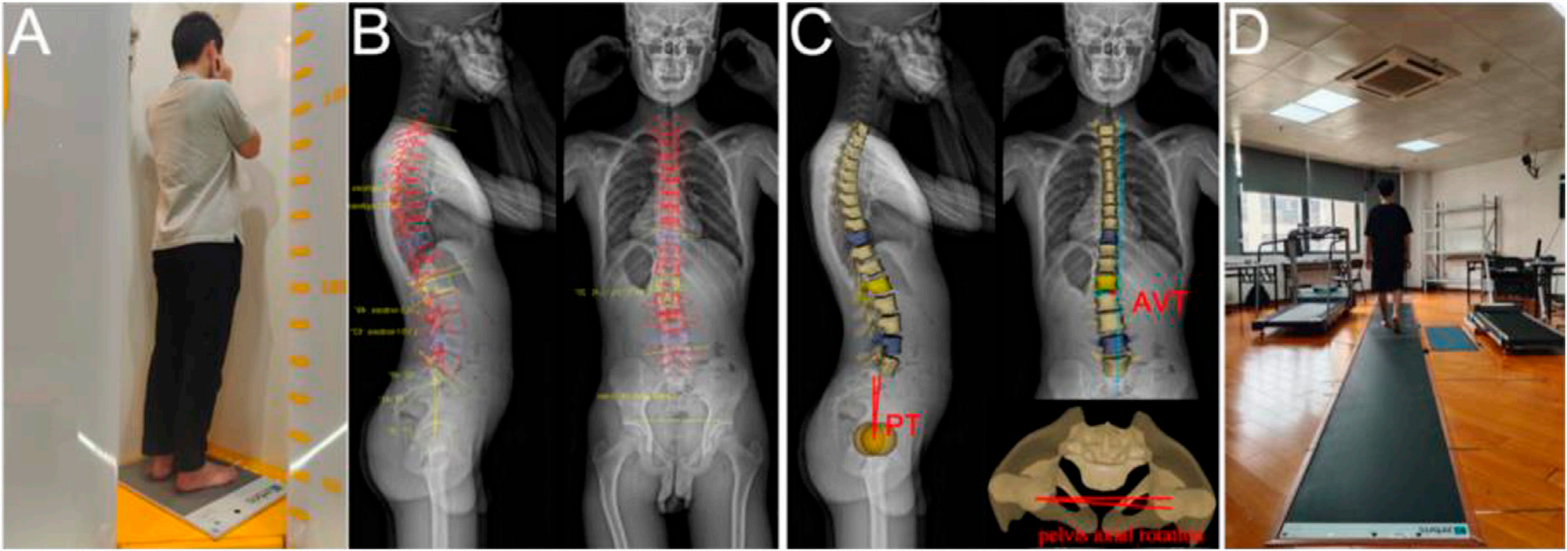
**(A)** is a Patient undergoing EOS imaging while his balance data is collected. **(B)** and **(C)** are the full 3-D recondtruction images of the spine performed by the radiologist using a post-processing software of EOS. **(D)** shows the gaint information of a patient being collected.

In patients with suspected AIS, further examination was performed using a whole-spine biplanar X-ray system (EOS Imaging^®^, Paris, France). To reduce the potential risk of radiation, posteroanterior views of the upper body and pelvis were taken using the micro-dose method. Subjects were asked to stand in the center of the cabin, with the pelvis located at the isocenter of the platform, and place both fists next to the cheeks with the upper arm at a 40° angle to the body and head facing forward ahead (Zhang et al., 2022). Simultaneously, a 0.5 m plantar pressure measurement mat (Zebris FDM, Germany) was placed under the patient’s foot to collect their static plantar center of pressure (CoP) information for 10 s (Figure 2). The following parameters were measured: the mean mediolateral CoP position (CoP_x_)—indicates the subject leaning laterally (the right side being positive), the mean anteroposterior CoP position (CoP_y_)—indicates the subject leaning anteroposteriorly (forward being positive), the CoP path length—the length of the subject’s CoP shift during the test, and the 90% confidence ellipse area—the area including 90% of all CoP points measured and transferred in the test. The center of the base of support (the area between the feet including the soles) is the origin for the CoP measurements. For the normal group, we acquired the aforementioned parameters using the same procedure but without the EOS examination. Afterward, a full 3D reconstruction of the spine was done by a single radiologist using a post-processing software for EOS (sterEOS^®^) (Courvoisier et al., 2013), which recognized anatomical landmarks and generated a 3D computer model of the full spine based on the synchronized posteroanterior and lateral images (Dubousset et al., 2007) (Figure 2B, C). The following radiographic parameters were collected: 1) coronal: Cobb’s angle, coronal balance, apical vertebra translation (AVT), and lateral pelvic tilt; 2) sagittal: thoracic kyphosis (TK), lumbar lordosis (LL), sagittal vertical axis (SVA), pelvic incidence (PI), sacral slope (SS), and sagittal pelvic tilt (PT); 3) axial: axial rotation of apical vertebra, pelvis axial rotation.

Then, all participants were required to walk barefoot through a 6-m plantar pressure measurement mat (Zebris FDM, Germany) at their comfortable pace (Figure.2D). Data for the following spatiotemporal gait parameters were collected: gait speed, cadence, stride length, step width, stance phase, swing phase, maximum pressure curve, and gait line. Both static and dynamic pressure experiments were carried out three times, and the average value was used for statistical analysis.

### Statistical analysis

All statistical analyses were performed using SPSS (version 26.0; SPSS IBM Inc, Armonk, NY). The Shapiro–Wilk test was used to check data normality, and independent-samples t-tests were performed to assess the between-group differences in demographic characteristics, balance, and gait data. All values are presented as mean ± standard deviations (SD). A *p*-value of <0.05 was considered statistically significant.

Additionally, stepwise multiple linear regression analyses were performed to identify any correlation between the balance and radiographic parameters. The entry and removal criteria of the model were the probability of F-values < 0.05 and F > 0.10, respectively. Parameters with an adjusted *p*-value of <0.05 were reported.

## Results

We included 34 patients (30 females) with Lenke type V and an equal number of age and sex-matched normal teenagers in the study; both groups were comparable in terms of demographic data (Table 1). The Cobb’s angle of the patients who were finally included in our study ranged from 20° to 34°, and the severity was moderate. The spinal and pelvic 3D parameters of the AIS group were as follows: 1) coronal: mean Cobb’s angle: 25.0° (21.8°–28.2°), coronal balance: 1.2 ± 1.1 mm, AVT: 1.4 ± 0.7 mm, and lateral pelvic tilt: 4.1 ± 3.7 mm. 2) sagittal: TK: 13.2° ± 8.2°, LL: 37.6° ± 11.7°, SVA: −6.7 ± 13.0 mm, PI: 42.2° ± 8.4°, SS: 34.8° ± 8.1°, PT: 5.7° ± 7.1°. 3) axial: axial rotation of apical vertebra: 7.2° ± 2.7° and pelvis axial rotation: 5.9° ± 3.2°.

**TABLE 1:** Demographic characteristics of study participants (*n* = 34).

Parameters	AIS	Normal	p
Age (years)	13.3 ± 2.2	14.1 ± 1.6	0.315
Height (cm)	162.5 ± 7.3	159.4 ± 4.4	0.203
Weight (kg)	47.5 ± 5.3	47.7 ± 3.8	0.900
BMI (kg/m2)	17.9 ± 0.9	18.8 ± 1.3	0.065

BMI, body mass index

We also observed a lower CoP_x_, a higher CoP path length and 90% confidence ellipse area in the AIS than the normal group. There was no statistically significant difference in the CoP_y_ value (Table 2).

**TABLE 2:** Static balance parameters in adolescent idiopathic scoliosis (AIS) and normal subjects (*n* = 34).

Parameters	AIS	Normal	p
CoPx (mm)	–13.7 ± 5.7*	–7.0 ± 5.4	0.029
CoPy (mm)	–28.8 ± 17.9	–22.4 ± 12.9	0.313
CoP path length (mm)	147.4 ± 58.1*	78.8 ± 32.0	0.008
90% confidence ellipse area (mm2)	150.5 ± 62.8*	92.1 ± 41.7	0.004

* means *p* < 0.05.

**FIGURE 3:**
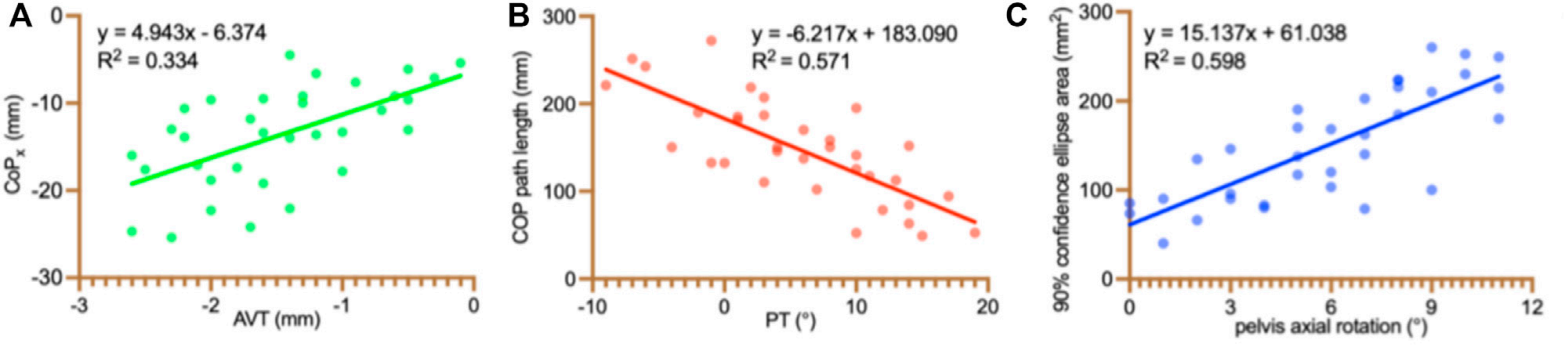
Statistically significant relationships between static balance and radiographic parameters. CoP: center of pressure; CoPx: the mean mediolateral CoP position; AVT: apical vertebra translation; PT: Sagittal pelvic tilt.

As per the results of the linear regression analysis for CoP_x_ and radiographic parameters, AVT was the only remaining variable in the regression analysis, accounting for 33.4% of the variance observed in CoP_x_ (Figure 3A). Likewise, the linear regression analysis of CoP path length and radiographic parameters revealed that PT was the only remaining variable, leading to a 57.1% variance observed in the CoP path length (Figure 3B). Regarding the 90% confidence ellipse area and radiographic parameters, pelvis axial rotation was the only remaining variable in the regression analysis, accounting for 59.8% of the variance observed in the 90% confidence ellipse area (Figure 3C). Lastly, in the linear regression analysis of CoP_y_ and radiographic parameters, no variable was remaining.

**FIGURE 4:**
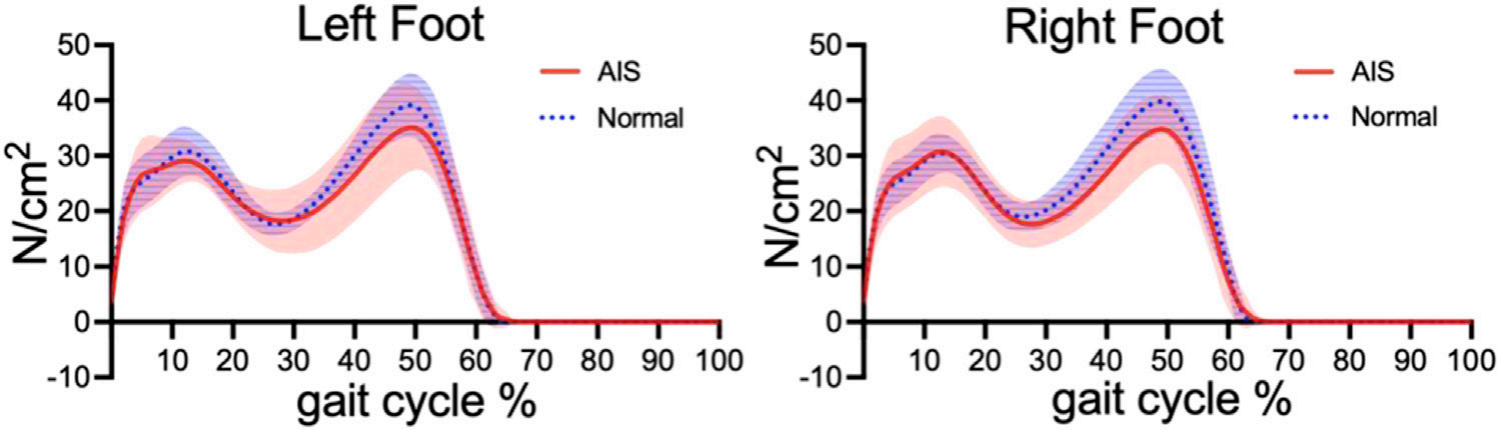
Bipedal maximum pressure curves of the two groups during gait cycle.Red indicates Adolesce Idiopathic Scoliosis (AIS) group and Blue indicates normal group. The solid line indicates th maximum pressure curve, and the shaded area is the standard deviation. Expect for the first peak of the right foot, the peak value of AIS group is lower than that of the normal group.

**FIGURE 5:**
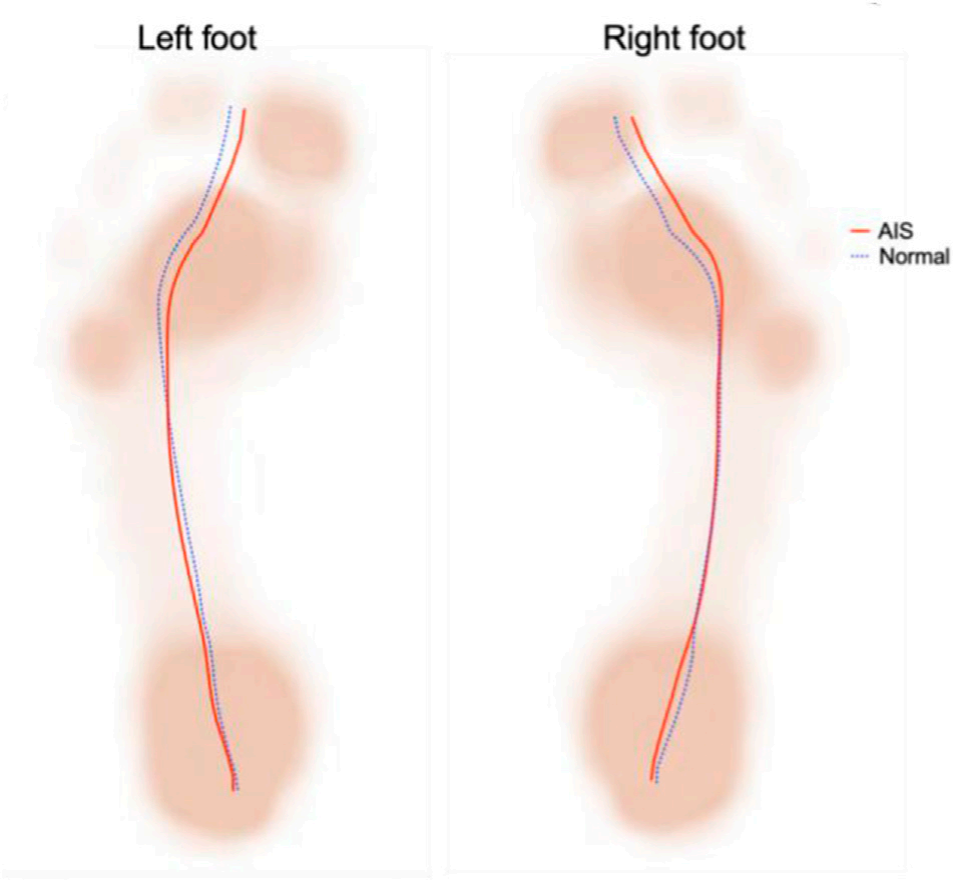
The gait line in Adolescent Idiopathic Scoliosis (AIS) and normal groups.Obviously ,the two groups are different, and bipedal asymmetry and Inconsistent change trend.

Regarding the gait parameters, the stance phase formed 61.35 ± 0.97% and 62.39 ± 1.09% of the total gait cycle in the AIS and normal groups, respectively, i.e., the stance phase was significantly reduced in AIS patients compared with the normal group, and the swing phase was correspondingly increased (Table 3). The two maximum pressure peaks of the left foot were smaller in the AIS group than those in the normal group, while that of the right foot was smaller only around the second peak (Figure 4). Figure 5 shows a representation of the gait line for bilateral plantar CoP progressed from the heel to the toe region between the normal and AIS groups. Compared to the normal group, the CoP trajectory of the left foot in the AIS group was initially on the lateral side of the normal group, then transitioned to the medial side gradually, and terminated at the medial side of the normal group. The right foot was just the opposite, i.e., initiating on the inside but ending on the outside.

**TABLE 3:** Gait parameters in adolescent idiopathic scoliosis (AIS) and normal subjects (*n* = 34).

Parameters	AIS	Normal	p
Gait speed (km/h)	4.41 ± 0.37	4.13 ± 0.49	0.149
Cadence (step/min)	115.20 ± 5.95	112.54 ± 5.99	0.301
Stride length (cm)	128.62 ± 12.55	122.19 ± 12.03	0.226
Step width (cm)	9.58 ± 2.56	11.19 ± 1.93	0.099
Stance phase (%)	61.35 ± 0.97*	62.39 ± 1.09	0.027
Swing phase (%)	38.66 ± 0.97*	37.62 ± 1.08	0.027

* means p < 0.05; The stance phase and the swing phase represent the proportion of the stance phase and the swing phase in the gait cycle, respectively.

## Discussion

In this study, we observed that the static balance of AIS patients was related to certain three-dimensional spinopelvic parameters, namely AVT, PT, and pelvic axial rotation. Although these Lenke V AIS patients primarily had lumbar scoliosis, the deformity at this level is anatomically related to the pelvis (Mac-thiong et al., 2006), which is an important determinant of lower limb loading pattern. It has been established that abnormal lower limb loading pattern ultimately leads to reduced balance (Márkus et al., 2018). Although many studies have explored the correlation between balance and spinopelvic parameters, most of these studies are limited to a single anatomical plane (Luo et al., 2021) (Ma, 2019) (Burkus et al., 2018); in contrast, we included spinopelvic parameters from all three dimensions. Luo et al. also recorded that PT was positively correlated with almost all baropodometric parameters on the major curve, but not with other parameters (Luo et al., 2021). Therefore, it can be inferred that PT best reflects the influence of sagittal plane balance on the lower limb. A previous study reported that the scoliosis group had a more significant correlation between standing stability and body posture parameters than the non-scoliosis group (Nault et al., 2002). Our innovative study procedure allowed for the synchronous acquisition of three-dimensional spinopelvic and standing stability parameters. We found that the correlation was multidimensional, i.e., static balance parameters are not only correlated with coronal and sagittal plane parameters, but also with axial plane parameters, which partially supports our study hypothesis. To the best of our knowledge, our results reveal characteristic associations that have rarely been elucidated in previous studies.

Our patients had an increased CoP path length and 90% confidence ellipse area as compared to the normal group, indicating poor static balance. Previous studies have also reported similar findings (Wiernicka, 2019). The CoP_x_ value was negative in our study, meaning the patients mostly leaned to the left. Catan et al. evaluated 32 girls diagnosed with idiopathic left convex lumbar curves and noticed a significantly higher load on the left foot (Cațan et al., 2020). The predominance of this left-sided imbalance can be attributed to the fact that all of our AIS patients had a thoracolumbar curve, which, anatomically, continues to show a left offset of the trunk and a right offset of the midline of the pelvis in the opposite direction, thus leading to a coronal postural imbalance. Interestingly, our study also highlighted the role of pelvic axial rotation on static balance disturbance, which has been rarely targeted in previous research (Luo et al., 2021) (Ma, 2019). Pelvis axial rotation is the result of the compensation for lumbar scoliosis, and the rotated pelvis is used as an unstable base of support for spine. The more severe the pelvic rotation, the greater the instability and the worse the balance. Notably, the three-dimensional spinopelvic and static balance parameters investigated simultaneously in our AIS patients significantly reduced the potential risks of inconsistent experimental procedures.

Another important finding of our study was the significantly reduced stance phase and a marked increase in the swing phase in AIS patients as compared with the normal group. However, there was no statistically significant between-group difference regarding the gait speed, cadence, stride length, and step width. The results are comparable to the findings of a previous meta-analysis by Kim et al. (Kim et al., 2020). These gait cycle alterations may be related to the significant increase in the electrical activity durations of the quadratus lumborum, erector spinae, and gluteus medius in the AIS group (Mahaudens, 2009); however, the exact mechanism underlying this process are yet to be explored.

The CoP gait line is a curve that directly reflects the nervous control of the ankle-foot complex muscles during walking (Tochigi, 2011) and also reveals the loading pattern and stability in the movement (Teyhen, 2009). We found that the CoP gait line of AIS patients was different from that of healthy individuals, indicating an ankle joint inversion or eversion transition during the stance phase that may lead to inefficient contact loading absorption, ankle injury, and falls. Gao et al. also described similar results (Gao, 2019). The lower peak of the maximal pressure curve in AIS patients compared to healthy individuals, especially in the single stance phase (between the two peaks), may be related to the lower arch in AIS patients, which was more pronounced in the left foot.

Based on these results, it can be recommended that the treatment and management of AIS patients should not only focus on the three-dimensional spinal alignment correction, but also gait and balance dysfunctions. Excellent balance can complete the movement safely and efficiently. Otherwise, the performance of ideal balance and symmetrical gait has a positive effect on maintaining the same activation of bilateral muscles, which may mitigate the deterioration of the curve. These findings further corroborate the importance of monitoring the balance and gait characteristics in AIS patients to understand the progress of the spinal curve.

There were certain limitations to this study. Firstly, AIS patients with Lenke V may be one of the most significant variations in the spinopelvic parameters. Therefore, the characteristics of other types are expected to be clarified by further research. Secondly, the sample size is relatively small, so the heterogeneity of curve severity is not carefully considered. Finally, gait parameters showed differences between the two groups, but the exact causes and mechanisms could not be deciphered from this cross-sectional study. Accordingly, large-scale, multi-centric, and well-designed studies are necessary to provide evidence to devise a more comprehensive clinical treatment for AIS patients.

## Conclusion

The standing balance of patients with Lenke V AIS is influenced by their AVT, PT, and pelvis axial rotation. We were able to record important differences in gait spatiotemporal parameters, maximum pressure curve, and gait line between AIS patients and age and sex-matched healthy subjects. We believe these findings may contribute to understanding the etiology of AIS and devising targeted comprehensive treatments.

## Data Availability

The raw data supporting the conclusion of this article will be made available by the authors, without undue reservation.
