# Supplementary material for: Correlational analysis of three-dimensional spinopelvic parameters with standing balance and gait characteristics in adolescent idiopathic scoliosis: A preliminary research on Lenke V
Source: Front Bioeng Biotechnol. 2022 Nov 30;10:1022376. doi: 10.3389/fbioe.2022.1022376 (PMC9747941; doi:10.3389/fbioe.2022.1022376)
Supplement: Supplementary file 1 [file DataSheet1.PDF]

Supplementary Material

1 Supplementary Figures and Tables

1.1 Supplementary Figures

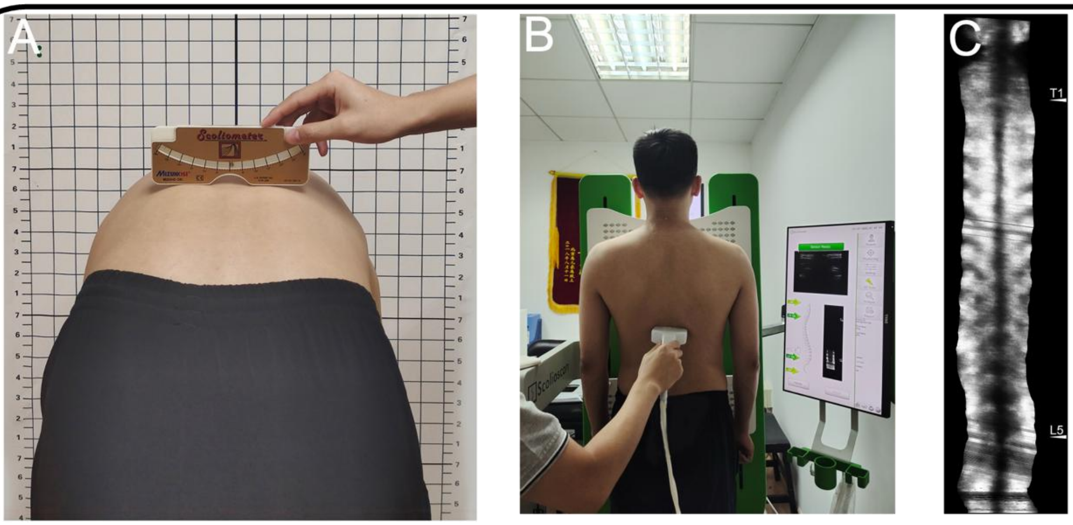

Fig.**1:** Scoliosis screening methods – (A) Adam's test and trunk rotation angle measured with a Scoliometer; (B) A radiation-free spine ultrasound imaging system; (C) Ultrasound imaging.

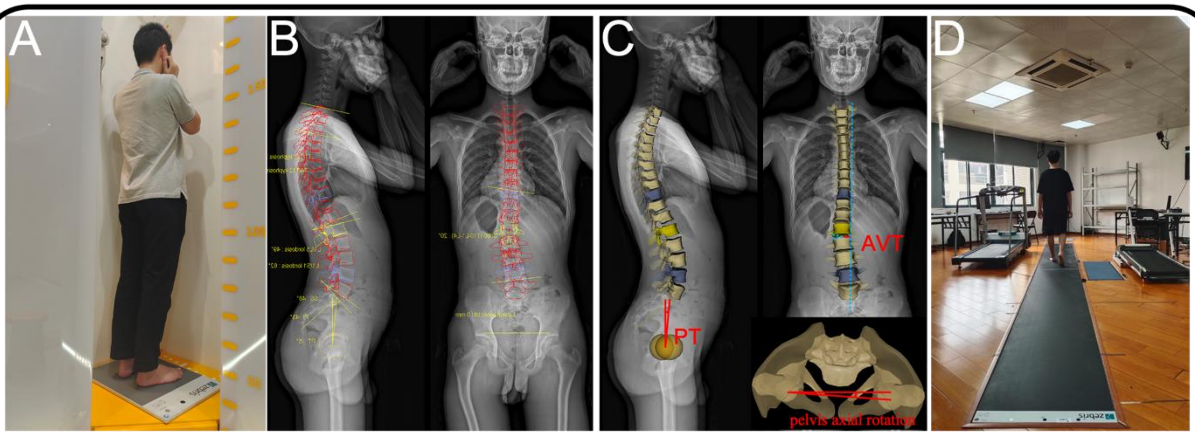

Fig.**2:** A is a patient undergoing EOS imaging while his balance data is collected. B and C are the full 3-D reconstruction images of the spine performed by the radiologist using a post-processing software of EOS. D shows the gait information of a patient being collected.

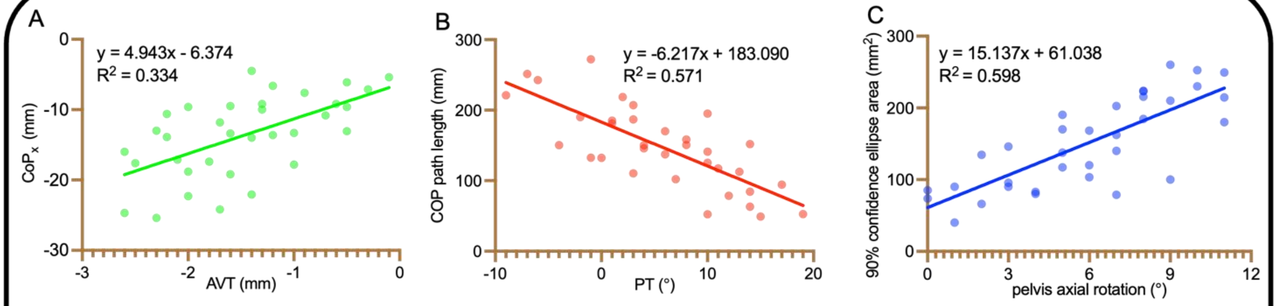

Fig.**3:** Statistically significant relationships between static balance and radiographic parameters. CoP: center of pressure; CoP_x: the mean mediolateral CoP position; AVT: apical vertebra translation; PT: sagittal pelvic tilt.

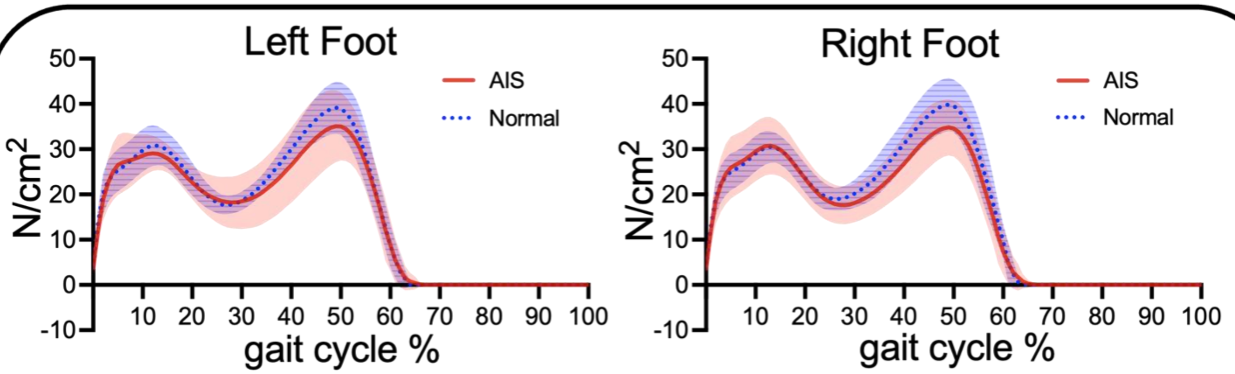

Fig.**4:** Bipedal maximum pressure curves of the two groups during gait cycle. Red indicates Adolescent Idiopathic Scoliosis (AIS) group, and blue indicates normal group. The solid line indicates the average maximum pressure curve, and the shaded area is the standard deviation. Except for the first peak of the right foot, the peak value of AIS group is lower than that of the normal group.

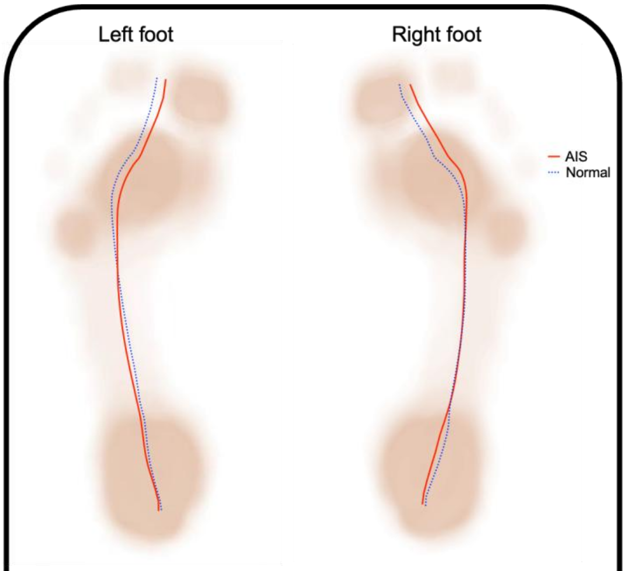

Fig.**5:** The gait line in Adolescent Idiopathic Scoliosis (AIS) and normal groups. Obviously, the two groups are different, and bipedal asymmetry and inconsistent change trend.

1.2 Supplementary Tables

Table1. Demographic characteristics of study participants (n=34)

**Parameters**	**AIS**	**Normal**	***p***
Age (years)	13.3±2.2	14.1±1.6	0.315
Height (cm)	162.5±7.3	159.4±4.4	0.203
Weight (kg)	47.5±5.3	47.7±3.8	0.900
BMI (kg/m^2)	17.9±0.9	18.8±1.3	0.065

BMI: body mass index.

Table 2. Static balance parameters in Adolescent Idiopathic Scoliosis (AIS) and normal subjects (n=34)

Parameters	AIS	Normal	*p*
CoP_x (mm)	-13.7±5.7*	-7.0±5.4	0.029
CoP_y (mm)	-28.8±17.9	-22.4±12.9	0.313
CoP path length (mm)	147.4±58.1*	78.8±32.0	0.008
90% confidence ellipse area (mm^2)	150.5±62.8*	92.1±41.7	0.004

* means $P<0.05$;

Table 3. Gait parameters in Adolescent Idiopathic Scoliosis (AIS) and normal subjects (n=34)

Parameters	AIS	Normal	*P*
Gait speed (km/h）	4.41±0.37	4.13±0.49	0.149
Cadence (step/min)	115.20±5.95	112.54±5.99	0.301
Stride length (cm)	128.62±12.55	122.19±12.03	0.226
Step width (cm)	9.58±2.56	11.19±1.93	0.099
Stance phase (%)	61.35±0.97*	62.39±1.09	0.027
Swing phase (%)	38.66±0.97*	37.62±1.08	0.027

* means P<0.05; The stance phase and the swing phase represent the proportion of the stance phase and the swing phase in the gait cycle, respectively.
